# CD45+ Cells Present Within Mesenchymal Stem Cell Populations Affect Network Formation of Blood-Derived Endothelial Outgrowth Cells

**DOI:** 10.1089/biores.2014.0029

**Published:** 2015-01-01

**Authors:** Erica B. Peters, Nicolas Christoforou, Erika Moore, Jennifer L. West, George A. Truskey

**Affiliations:** ^1^ Department of Biomedical Engineering, Duke University, Durham, North Carolina.; ^2^ Department of Biomedical Engineering, Khalifa University, Abu Dhabi, United Arab Emirates.; ^3^ Department of Mechanical Engineering and Materials Science, Duke University, Durham, North Carolina.; ^4^ Department of Cell Biology, Duke University, Durham, North Carolina.; ^5^ Department of Chemistry, Duke University, Durham, North Carolina.

**Keywords:** angiogenesis, vasculogenesis, endothelial progenitor cells, mesenchymal stem cells, umbilical cord blood, microvessel tissue engineering

## Abstract

Mesenchymal stem cells (MSCs) and endothelial progenitor cells (EPCs) represent promising cell sources for angiogenic therapies. There are, however, conflicting reports regarding the ability of MSCs to support network formation of endothelial cells. The goal of this study was to assess the ability of human bone marrow-derived MSCs to support network formation of endothelial outgrowth cells (EOCs) derived from umbilical cord blood EPCs. We hypothesized that upon *in vitro* coculture, MSCs and EOCs promote a microenvironment conducive for EOC network formation without the addition of angiogenic growth supplements. EOC networks formed by coculture with MSCs underwent regression and cell loss by day 10 with a near 4-fold and 2-fold reduction in branch points and mean segment length, respectively, in comparison with networks formed by coculture vascular smooth muscle cell (SMC) cocultures. EOC network regression in MSC cocultures was not caused by lack of vascular endothelial growth factor (VEGF)-A or changes in TGF-β1 or Ang-2 supernatant concentrations in comparison with SMC cocultures. Removal of CD45+ cells from MSCs improved EOC network formation through a 2-fold increase in total segment length and number of branch points in comparison to unsorted MSCs by day 6. These improvements, however, were not sustained by day 10. CD45 expression in MSC cocultures correlated with EOC network regression with a 5-fold increase between day 6 and day 10 of culture. The addition of supplemental growth factors VEGF, fibroblastic growth factor-2, EGF, hydrocortisone, insulin growth factor-1, ascorbic acid, and heparin to MSC cocultures promoted stable EOC network formation over 2 weeks *in vitro*, without affecting CD45 expression, as evidenced by a lack of significant differences in total segment length (*p*=0.96). These findings demonstrate the ability of MSCs to support EOC network formation correlates with removal of CD45+ cells and improves upon the addition of soluble growth factors.

## Introduction

Microvessels are critical for the healthy function of all organs in our body through the delivery of oxygen to tissues and removal of metabolic waste.^1^ In addition, regulation of microvessel development is essential for the management of several disease types.^2^ Recent studies have demonstrated the ability of mesenchymal stem cells (MSCs) to serve as pericyte-progenitor cells, revealing novel opportunities for the use of MSCs in vascular therapies.^3–6^ For example, human bone marrow–derived MSCs enhance early stages of angiogenesis *in vitro* through upregulation of angiogenesis-associated genes, such as vascular endothelial growth factor (VEGF) and matrix metalloproteinases, allowing endothelial cells (ECs) to migrate and elongate.^7–12^ These *in vitro* observations of MSCs' function as mural cells are extended *in vivo* where MSCs combined with endothelial outgrowth cells (EOCs) derived from umbilical cord blood endothelial progenitor cells (EPCs) within a Matrigel^™^ system and implanted in a murine model demonstrated perivascular localization and supported EOC vascular networks for 4 weeks post implantation.^7,13^

However, there exist reports identifying the anti-angiogenic potential of MSCs.^14–17^ For example, MSCs added to preformed EC networks within an *in vitro* Matrigel^™^ system increased the production of reactive oxygen species, resulting in EC network regression and apoptosis.^16^ Further, MSC injection to preformed microvessels within an *in vivo* murine tumor model inhibited angiogenesis by decreasing microvascular density.^16^ These contradictory results for the effect of MSCs upon EC network formation raise concerns in the clinical efficacy of utilizing MSCs for angiogenic therapies.

The conflicting pro- and anti-angiogenic effects of MSCs upon ECs may be due, in part, to the variability of conditions within *in vitro* and *in vivo* model systems of microvessel formation.^7,13,14^ The presence of additional cell types, supplemental growth factors, and biologically derived matrices vary between studies, confounding interpretations of MSC behavior. For instance, biologically derived gels containing collagen derivatives can engage a greater range of integrins than tissue-culture polystyrene substrates, potentially activating EC signaling pathways that promote microvessel formation.^18^ The absence of biologically derived extracellular matrix components, or angiogenic stimulating growth factors, may hinder the ability of MSCs to support EC network formation. In addition, conventional methods for MSC selection from bone marrow aspirates are based upon adherence to tissue culture plastic. This selection criteria, however, is not unique to MSCs and can result in coexpansion with additional adherent cell populations, such as macrophages.^19,20^ The absence of positive controls during fluorescently activated cell sorting (FACS) procedures to purify MSC populations may enable trace populations of proinflammatory, polynuclear CD45+ cells, such as macrophages, to escape detection, causing issues with the ability of MSCs to promote stable, robust network formation of ECs.

One approach to fully characterize the role of MSCs upon EC network formation is to employ a reductionist experimental system that compares observations of MSC effects on EC network formation against a positive control model of ECs and mural cells. The highly angiogenic ability of vascular smooth muscle cells (SMCs) renders them an appropriate positive control for evaluating the angiogenic potential of MSCs. SMCs have been shown to support stable, robust microvessel formation of ECs across a range of physiologically relevant elastic moduli under culture conditions that require minimal supplemental growth factors.^21–24^ EC networks derived from coculture with SMCs are observable for over one month *in vitro,* demonstrate lumen formation, and mimic *in vivo* physiological processes of angiogenesis by preventing continuous proliferation of ECs.^22–24^

Despite their promising pro-angiogenic function, SMCs are not a practical source of cells for large-scale fabrication of tissue engineered microvessels due to the additional donor-site morbidity associated with cell harvest and enhanced risk of immunogenicity in allogeneic transplants. MSCs represent a promising source of mural cells due to their SMC differentiation potential, immunoregulatory properties, and ease of isolation from several tissues.^2,3,8^ Furthermore, EOCs obtained from umbilical cord blood EOCs represent a promising allogenic EC source for angiogenic therapies.^7,9,11–13,23^ In this study, we evaluated the ability of MSCs to support EOC network formation through a reductionist, *in vitro* coculture model containing no additional growth factors beyond those present in serum. Network formation by EOCs in coculture with MSCs was observed over a 2-week period with an analysis of the effects of MSCs upon EOC network morphology, supernatant concentrations of angiogenic cytokines, and gene and protein expression of endothelial and mural cell markers. In addition, positive controls of CD45+ monocytes were employed during FACS of MSCs to evaluate the impact of potential macrophage populations on EOC network formation.

## Materials and Methods

### Cell culture and maintenance

Human bone marrow–derived MSCs used in this study were obtained from Lonza and also generously provided by Darwin J. Prockop of Texas A&M Institute for Regenerative Medicine. The MSCs were expanded in minimum essential medium alpha medium (Gibco) supplemented with 20% fetal bovine serum (FBS), 1% antibiotic–antimycotic solution, and 1% l-glutamine. MSCs were characterized according to the standards for defining MSCs set by the International Society of Cellular Therapy.^25^ MSCs were assessed for positive expression of markers CD90, CD105, CD73, and negative expression of CD45, CD34, CD14, CD19, and major histocompatibility complex, class II, DR alpha (HLA-DR) in comparison with a mouse immunoglobin G (IgG) isotype control using antibodies preconjugated to fluorescein isothiocyanate (FITC) or phycoerythrin (PE) (Biolegend) (Supplementary Fig. S1). StemPro^®^ differentiation kits (Invitrogen) were utilized to confirm osteogenic, adipogenic, and chondrogenic differentiation of MSCs (Supplementary Fig. S1). MSCs from two donors were used between passages 3 and 5.

Umbilical cord blood was obtained from the Carolina Cord Blood Bank following approval from the Duke University Institutional Review Board. EPCs were isolated using a previously established protocol.^26^ Mononuclear cells (MNCs) were plated at a density of 80×10^6^ cells per well onto six-well plates (BD Falcon) precoated with collagen 1 (BD Biosciences) and supplemented with endothelial basal media-2 (EBM-2, Lonza) containing endothelial growth media-2 SingleQuots (Lonza) with 10% FBS and 1% antibiotic–antimycotic solution. Endothelial colony-forming units were observed between 9 and 22 days after MNC plating, expanded, and confirmed for EOC phenotype through flow cytometry for expression of CD31 and CD34 (Supplementary Fig. S2) and formation of capillary-like networks within a Matrigel^™^ system (Supplementary Fig. S2).^27^ EOCs from three donors were used between passages 3 and 5.

Human aortic vascular smooth muscle cells (SMCs) were purchased from Cambrex and maintained in smooth muscle basal medium supplemented with smooth muscle growth media-2 SingleQuots (Lonza) and 1% antibiotic–antimycotic solution. SMCs from two donors were used at passages 5–8.

Human monocytes were purchased from ATCC and maintained in suspension culture with RPMI-1640 (ATCC) growth media containing 10% FBS, 1% antibiotic–antimycotic solution, and 50 μM of 2-mercaptoethanol.

### Characterization of EOC network formation within coculture systems

To form cocultures, EOCs (4.8×10^4^ cells/cm^2^) were mixed with 8×10^4^ cells/cm^2^ of MSCs or SMCs and plated on polystyrene 24-well plates (BD Falcon). These seeding densities were chosen using a previously established coculture system of SMCs and EOCs that resulted in extensive endothelial network formation.^24^ The coculture media consisted of EBM-2 supplemented with 10% FBS and 1% antibiotic–antimycotic solution. The media was changed every 48 hours.

To visualize the cells, EOCs were transduced with tdTomato-expressing lentivirus, while MSCs and SMCs were transduced with green fluorescent protein (GFP)-expressing lentivirus. The lentivirus vector system used packaging vectors psPAX2 (Addgene No. 12260) and pMD2G (Addgene No. 12259). For GFP expression, the vector FUGW (Addgene No. 14883) was used. For the flap-Ub promoter (FU) tdTomato.W, we replaced GFP in the FUGW with the tdTomato gene. The transduction efficiency was nearly 100% for the EOCs, MSCs, and SMCs. Images of cocultures were taken on a Nikon^®^ Eclipse Inverted Microscope system. Quantitative analysis of EOC network formation was performed using MetaMorph's Angiogenesis Tube Formation module with a maximum capillary width of 50 μm and a minimum width of 3 μm for EOCs in coculture and 13.1 μm for EOCs in monoculture.

To evaluate the viability of cells within MSC and SMC cocultures, we performed a live/dead assay (LIVE/DEAD^®^ Viability/Cytotoxicity Kit, Life Technologies) after 8 days of culture. Coculture media containing 4 μM ethidium homodimer-1 (EthD-1) and 4 mM calcein acetomethoxy solution were added to SMC and MSC cocultures and incubated for 2 hours prior to imaging. EthD-1 expression was quantified by calculating the raw integrated pixel density per image with FIJI software and normalized to the image area.

To characterize levels of angiogenic proteins within coculture conditions, supernatant samples were collected at time points of day 2, 6, 10, 14, and 18 after plating with media changes occurring 24 hours before each time point. Quantitative determination of VEGF-A, transforming growth factor (TGF)-β1, and angiopoietin-2 (Ang-2) levels was performed using Quantikine^®^ enzyme-linked immunosorbent assay (ELISA) (Nos. DVE00, DB100B, and DANG20, respectively, R&D Systems) according to the manufacturer's suggested protocol.

To evaluate whether mural cell and EC differentiation were present in MSC and EOC cocultures, quantitative real-time (qRT)-PCR was performed on the following markers associated with mural cell differentiation and EC differentiation after 18 days of culture: EC differentiation–VEGF receptor 2 (VEGFR-2),^1,28^ tyrosine kinase with immunoglobulin-like and EGF-like domains (Tie)-1, Tie-2,^29–31^ mural cell differentiation–myosin heavy chain 11 (MYH-11), platelet-derived growth factor β receptor (PDGF β-R), and chondroitin sulfate proteoglycan-4 (CSPG-4).^1,32^ The housekeeping gene was 18s ribosomal RNA. Total RNA (50 ng) was reverse transcribed using the cDNA Synthesis Kit (Bio-Rad) and a MyCycler (Bio-Rad) thermal cycler. Primers (Integrated DNA Technologies) were confirmed for specificity to targets using conventional PCR. The sequences for each primer are listed in Supplementary Table S1. The 2(-Delta DeltaC (T)) method was used to determine relative gene expression to SMCs.

To further evaluate mural cell and EC differentiation, immunofluorescence for protein expression of alpha-smooth muscle actin (α-SMA), PDGFβ-R, and platelet endothelial cell adhesion molecule (PECAM/CD31) was performed on MSC and EOC cocultures. Cocultures were plated on 35-mm glass dishes (FluoroDishTM, World Precision Instruments, Inc.) precoated with 3.3 μg/mL of fibronectin for 1 hour. After 10 days of culture, samples were fixed in methanol for 3 minutes and blocked in a 10% solution of goat serum in phosphate-buffered saline (PBS) for 1 hour. Primary antibodies were diluted with blocking solution [α-SMA 1:100 (Abcam), PDGFR-β 1:50 (Santa Cruz), NG2/CSPG-4 1:50 (Santa Cruz), TIMP-3 1:1000 (Abcam), PECAM 1:100 (Invitrogen)] and incubated with the cultures at 4°C for 5 hours. Samples were washed with PBS and incubated with the appropriate secondary antibody [Alexa Fluor 488 or Alexa Fluor 594 1:1000 (Invitrogen)] overnight. After another wash with PBS, samples were incubated with a 5 μg/mL DAPI solution for 30 minutes in order to visualize nuclei. The cultures were imaged on a Leica SP5 confocal microscope at 20× magnification, 1280×1280 resolution with a line scanning average of 6.

### Evaluation of CD45+ cells, within MSC populations, effect upon EOC network formation

Supernatant samples of MSC and SMC cocultures were collected at time points of day 2, 6, 10, 14, and 18 after plating with media changes 24 hours before each time point and measured for inflammatory markers interferon (IFN)-γ, IFN-α, and thrombospondin (TSP)-1^33–39^ through ELISA assays (R&D systems) according to the manufacturer's suggested protocol.

To investigate whether phagocytosis occurred in EOC and MSC cocultures, we performed a phagocytosis assay over the first 10 days of coculture (Vybrant^®^ Phagocytosis Assay Kit, Life Technologies). *Escherichia coli*, fluorescently tagged with fluorescein, was added to MSC cocultures at day 2, 6, and 10 of coculture and allowed to incubate for 2 hours before quenching with trypan blue prior to analysis. Murine macrophage cells (J774 cells, ATCC) and Dulbecco's modified Eagle's medium media containing no cells served as positive and negative controls, respectively. Fluorescence analysis was performed on an Infinite^®^ 200 Pro (TECAN) fluorescence plate reader at 480 nm excitation and 520 nm emission. The phagocytosis effect is the net experimental reading divided by the net positive reading. The net experimental reading is the fluorescence intensity of the negative-control wells subtracted from the experimental wells. The net positive reading is the fluorescence intensity of negative-control wells subtracted from positive control wells.

### Fluorescently activated cell sorting

FACS was performed on MSCs against a monocyte positive control for CD45 to improve the purity of MSC populations. MSCs were incubated with CD45-FITC, Mouse IgG isotype control FITC (Biolegend)at a concentration of 2 μl per 1×10^5^ cells for 30 min at room temperature before resuspension in PBS. MSCs were centrifuged at 200 *g* and resuspended in culture media sorted for CD45- populations with gating for relative fluorescence intensity against CD45-FITC positive monocytes. CD45- MSCs were cocultured with EOCs using EBM-2 media supplemented with 10% FBS and 1% antibiotic–antimycotic solution and compared to unsorted MSC and SMC cocultures for effects on EOC network formation. Immunofluorescence for CD45, PECAM, and α-SMA was performed on days 2, 6, and 10 of coculture. Cultures were fixed and imaged following methods described for characterization of EOC network formation with. CD45 (Abcam) added at a 1:500 dilution in 10% goat serum in PBS. Quantification of CD45+ cells within cocultures was done through integrated pixel density normalized to the total number of cells per image area using FIJI software.

### Statistical analysis

All data are presented as means and standard errors with differences among coculture conditions established by ANOVA using factors of coculture condition, day, and time. Post-hoc tests for individual treatment effects were done using a Student's *t*-test. A *p*-value of less than 0.05 was used to indicate significance. For the qRT-PCR studies, the global intensity normalization method was performed to account for variation among donors.^40^ To correct for donor variability, the normalization factor was calculated as the ratio of average mRNA expression for each condition to the global average cDNA expression of all conditions. After confirming the variance of the qRT-PCR samples as nonhomogeneous by the Welch's test for unequal variances, nonparametric testing was performed using the Kruskal-Wallis Test. JMP^®^ 9.0 software was used for all statistical analysis.

## Results

### In the absence of supplemental growth factors, EOCs undergo regression and cell loss in MSC cocultures

**FIG. 1.**
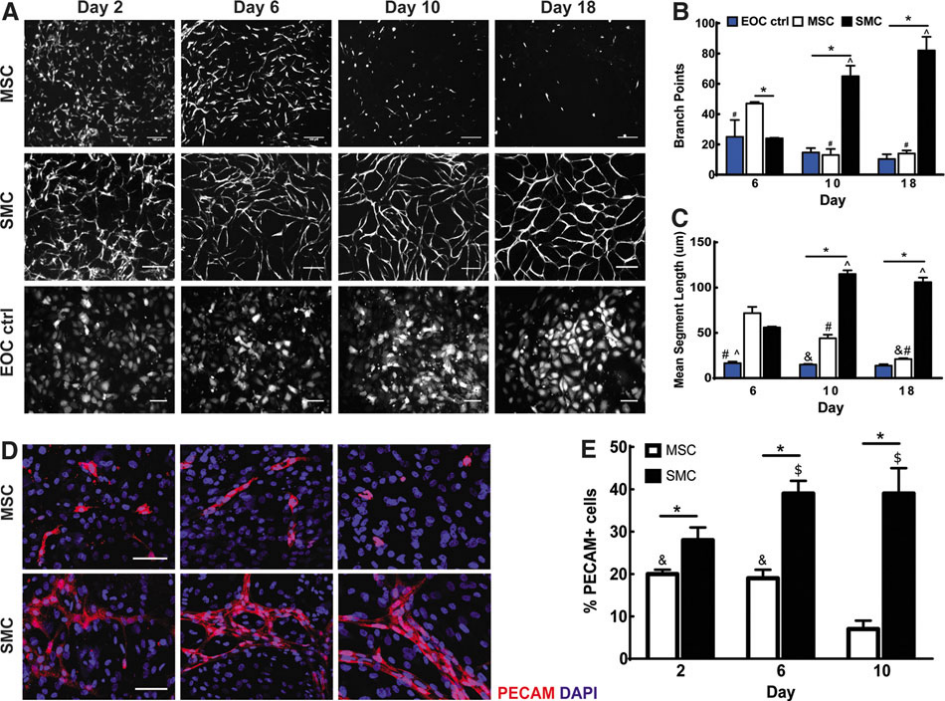
Comparison of mesenchymal stem cell (MSC) and smooth muscle cell (SMC) coculture upon endothelial outgrowth cell (EOC) network formation *in vitro*. Images are representative of three independent experiments. Scale bars equal 100 μm. **(A)** EOCs were transduced with tomato-expressing lentivirus and combined with either MSCs or SMCs for observation over 18 days of coculture. Changes in EOC network morphology between MSC and SMC coculture conditions over time were quantified through analysis of the number of branch points **(B)** and mean segment length **(C)**. **(D, E)** EOCs in MSC cocultures underwent cell loss over 10 days of culture in contrast to SMCs as evidenced by the percentage of platelet endothelial cell adhesion molecule (PECAM)–positive cells, normalized to total cell number. Image area analyzed is 0.57 mm^2^ with *n*=3 images analyzed per condition. *Indicates *p*<0.05, ^#^indicates *p*<0.05 in comparison to Day 6 of MSC coculture, ^^^indicates *p*<0.05 in comparison to Day 6 of SMC coculture, ^$^indicates *p*<0.05 in comparison to Day 2 of SMC coculture, ^&^indicates *p*<0.05 in comparison to day 10 of MSC coculture.

To determine whether MSCs are able to function as mural cells by supporting stable, robust EOC network formation *in vitro*, we cocultured MSCs and EOCs using conditions previously determined to support EOC network formation while being cocultured with SMCs,^24^ over a period of 18 days (Fig. 1A). MSCs supported initial migration and elongation of EOCs achieving a 2-fold increase in average segment length in comparison to SMC cocultures by day 6 of culture. However, the EOC network structures were not stable, undergoing regression and cell loss by day 10 of culture as evidenced by a near 4-fold decrease in the number of branch points and significant decreases in average segment length (*p*=0.0007) and percentage of PECAM-positive cells (*p*=0.015) in comparison with day 6 of culture (Fig. 1A, B). In contrast, cocultures of SMCs and EOCs demonstrated significant increases in branch points (*p*<0.0001) and average segment length (*p*<0.0001) between days 6 and 10 and cell number (*p*=0.0193) between days 2 and 6, which were maintained through day 18 of culture. Both the SMCs and MSCs were dispersed throughout the coculture system at day 10 of culture, excluding the possibility of MSC loss contributing to EOC regression (Supplementary Fig. S3) . We observed a significant increase in EthD-1 expression, an indicator cell death, in MSC cocultures in comparison to SMC cocultures (*p*=0.01) at day 8 of culture (Supplementary Fig. S4). The lack of supplemental endothelial growth factors, such as VEGF, within the coculture media did not cause EOC loss as evidenced through EOC monoculture controls (Fig. 1A, B). These observations demonstrate that under these culture conditions, MSCs are not unable to support long-term EOC network formation to the same extent as SMCs, and instead cause EOC loss.

### MSC cocultures contain higher levels of VEGF-A than SMC cocultures and demonstrate gene expression of mural cell markers

Due to the complexity of processes involved in angiogenesis,^1^ there are several factors that may contribute to the impaired ability of MSCs to promote network formation of EOCs observed in Fig. 1. For example, soluble growth factors serve a critical role in angiogenesis with VEGF acting as a key regulator in the early stages of angiogenesis, initiating endothelial cell migration and tube formation.^1^ Latent forms of transforming growth factor beta 1 (TGF-β1) produced by ECs become activated upon contact with mural cells, serving to differentiate the recruited cells into pericytes, which act to stabilize the newly formed capillary networks by preventing apoptosis.^1,41^ Overexpression of Ang-2 disrupts pericyte-EC contacts, leading to EC network regression and apoptosis.^42,43^ We hypothesized the impaired ability of MSCs to promote EC network formation may be due to differences in levels of angiogenic proteins VEGF, TGF-β1, and Ang-2 in comparison with SMC cocultures. To test our hypothesis, supernatant was collected from MSC and SMC cocultures over 18 days of culture and analyzed using ELISA.

**FIG. 2.**
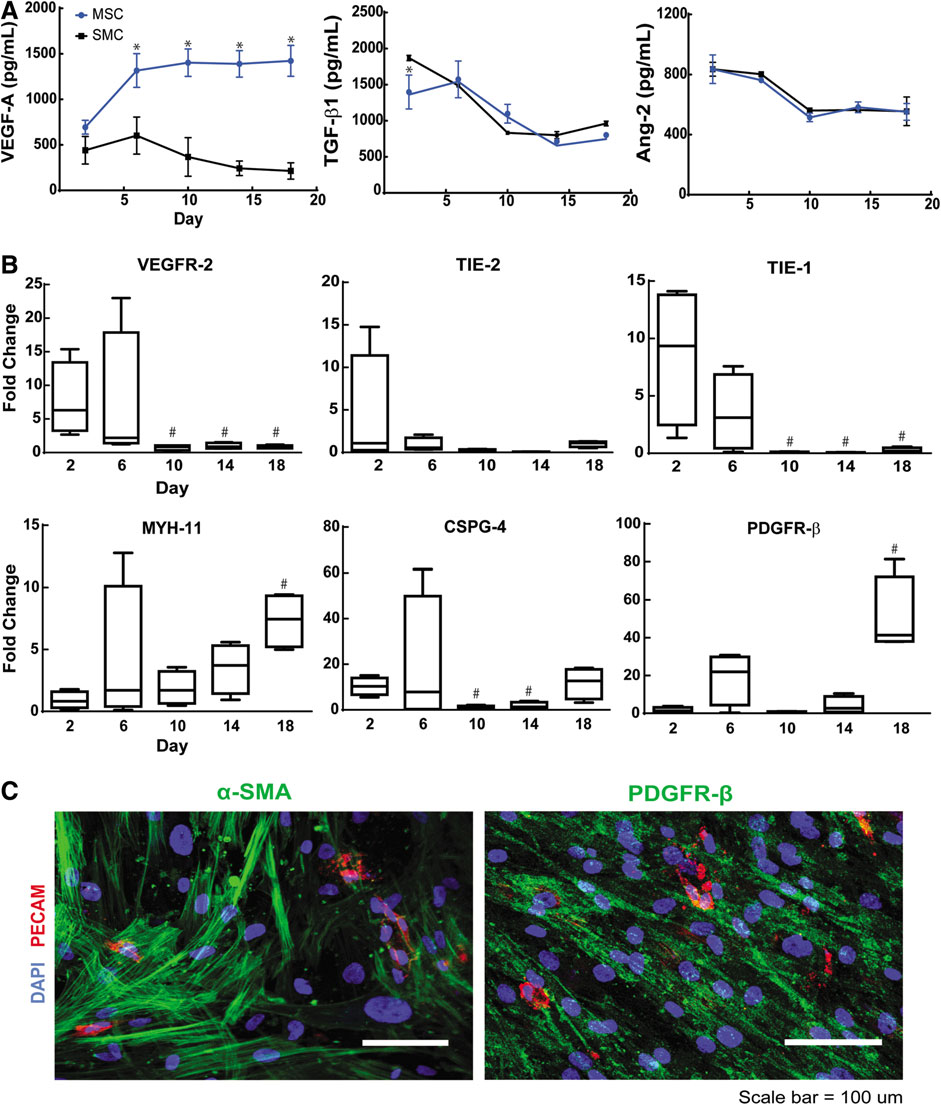
**(A)** Measured supernatant levels for vascular endothelial growth factor (VEGF)-A, transforming growth factor (TGF)-β1, Ang-2 in cocultures of EOCs with MSCs or SMCs during the first 18 days of culture. *Indicates *p*<0.05 in comparison to SMC conditions at the same day of coculture as MSC conditions; *n*=3 supernatant samples analyzed per time point, per coculture condition. **(B)** Fold difference in RNA transcription levels of MSC cocultures relative to SMCs. ^#^Indicates *p*<0.05 in comparison to day 2 MSC cocultures within the gene group analyzed; *n*=4 RNA transcription levels analyzed per time point, per gene analyzed. **(C)** Immunofluorescence at day 10 of MSC coculture for α-smooth muscle actin (α-SMA), PDGF receptor beta (PDGFR-β), and platelet endothelial cell adhesion molecule (PECAM). Scale bar equals 100 μm.

We observed a nearly 2-fold increase in VEGF-A supernatant levels in MSC cocultures in comparison with SMC cocultures which persisted through day 18 of culture (Fig. 2A). In contrast, supernatant levels were nearly identical between MSC and SMC cocultures for Ang-2 (*p*=0.972) and showed no significant difference in TGF-β1 after day 2 of culture (Fig. 2A). These findings indicate impaired EOC network formation within MSC cocultures *in vitro* is not due to decreased VEGF-A, TGF-β1 or increased Ang-2 protein levels in comparison to SMC cocultures.

Co-cultivation of EOCs and MSCs can increase gene expression of mural cell markers such as α-SMA.^11,44^ Incomplete differentiation of MSCs towards an SMC phenotype may result in impaired angiogenic function. MSCs have also been shown to have the potential to differentiate towards an EC lineage.^45,46^ While EOCs underwent cell loss by 10 days of culture (Fig. 1A, E), populations of MSCs within coculture may have differentiated towards an EC lineage in addition to a mural cell lineage. We performed qRT-PCR on EOC:MSC cocultures for gene expression of mural cell markers CSPG-4, PDGFR-β, and MYH-11 and EC markers associated with angiogenesis: TIE-2, TIE-1, and VEGFR-2 (Fig. 2B). After 18 days of coculture, we observed a 40-fold increase in PDGFR-β, 5-fold increase in MYH-11, and no change in CSPG-4 gene expression in comparison to SMCs. In contrast, we found a significant decrease in gene expression for TIE-1 (*p*=0.0304) and VEGFR-2 gene expression (*p*=0.0304) with no significant change in TIE-2 expression after 10 days of coculture. Immunofluorescence at day 10 of MSC coculture confirms the expression of α-SMA, PDGFR-β, and lack of ECs through PECAM staining (Fig. 2C).

The decrease in EC gene expression correlates to the loss of EOCs observed in coculture with MSCs. Our findings demonstrate agreement with previously reported findings of EOC and MSC coculture leading to increased gene expression of mural cell markers MYH-11 and PDGFR-β, over several days of culture. In addition, the loss of EOCs observed upon coculture with MSCs (Fig. 1) was not replaced by ECs differentiated from remaining MSC populations.

### The presence of CD45 expressing cells is correlated with the extent of EOC network formation in MSC and SMC cocultures

Based on our findings, the observed cell loss of EOCs in coculture with MSCs in comparison with SMC cocultures is not caused by a lack of VEGF-A or TGF-β1 or increase of Ang-2 protein levels in the supernatant, or the lack of differentiation towards a mural cell phenotype based on the increased gene expression of α-SMA and PDGFR-β over 2 weeks of coculture. Another explanation for EOC cell loss could be due to the presence of contaminating proinflammatory cells, such as macrophages, within the MSC population. The role of macrophages in angiogenesis is complex and dependent on the context of the local microenvironment.^47^ For example, in the presence of the cytokine interferon gamma (IFN-γ), macrophages are induced into a proinflammatory phenotype which possess phagocytic ability that may disrupt microvessel formation.^48^ In contrast, interleukin-4 stimulates macrophages towards a pro-angiogenic phenotype through the secretion of growth factors such as VEGF-A and basic fibroblastic growth factor (FGF-2).^48^

**FIG. 3.**
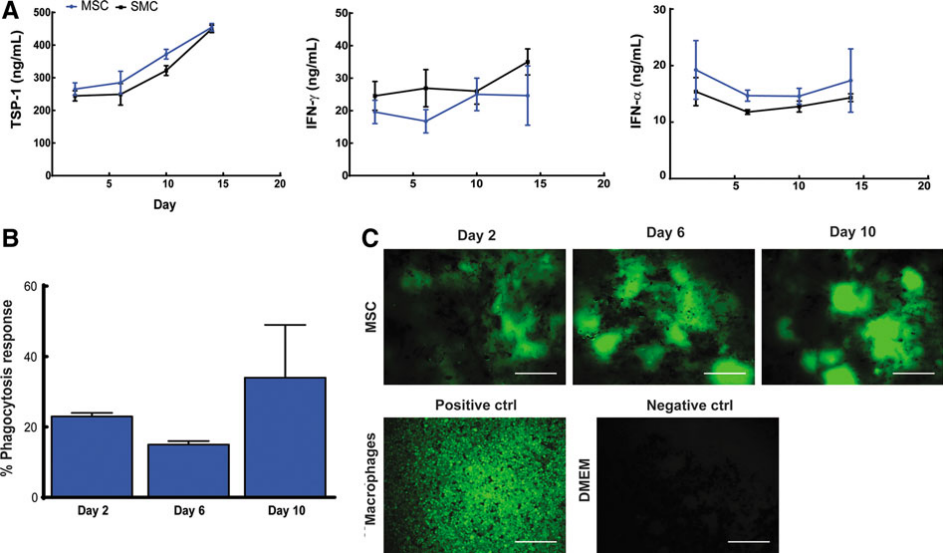
**(A)** Measured supernatant levels for thrombospondin-1 (TSP-1), interferon-gamma (IFN-γ), and interferon-alpha (IFN-α) in cocultures of EOCs with MSCs or SMCs during the first 18 days of culture. **(B)** Analysis of phagocytosis response within EOC and MSC cocultures during the first 10 days of culture; *n*=4 wells analyzed per condition. **(C)** Representative phagocytosis assay images depicting intracellular, fluorescein-labeled *Escherichia coli* within MSC cocultures during the first 10 days of coculture. Positive controls consisted of macrophages and negative controls consisted of experimental wells containing only Dulbecco's modified Eagle's medium. Scale bar equals 200 μm.

We tested the hypothesis that EOC regression and loss in MSC cocultures is caused by elevated levels of inflammatory cytokines IFN-γ, IFN-α, and thrombospondin (TSP-1), known to inhibit EC growth and angiogenic function.^40–46^ We found no significant increases in the levels of IFN-γ (*p*=0.77), IFN-α (*p*=0.97), or TSP-1 (*p*=0.90) in the supernatant from MSC cocultures in comparison to SMC cocultures during the first 18 days of culture (Fig. 3A). We also examined the contribution of phagocytosis to EOC loss and found evidence of phagocytic activity within MSC cocultures throughout the first 10 days of culture based upon uptake of fluorescent *E. coli* particles Fig. 3B).

Resident tissue macrophages may be present in trace amounts within isolated MSC populations.^49^ These macrophages could escape detection during FACS if a positive control for CD45+ expressing macrophages was not employed. We hypothesized CD45+ cells may represent potential macrophages present within the MSC population, which could contribute to EOC loss upon coculture and explain the observed phagocytic response. To test our hypothesis we performed FACS on MSCs, employing a positive control of CD45-expressing monocytes, and cocultured the resulting CD45- MSCs with EOCs. We compared the effect on EOC network formation against unsorted MSC and SMC cocultures over 2 weeks *in vitro*.

**FIG. 4.**
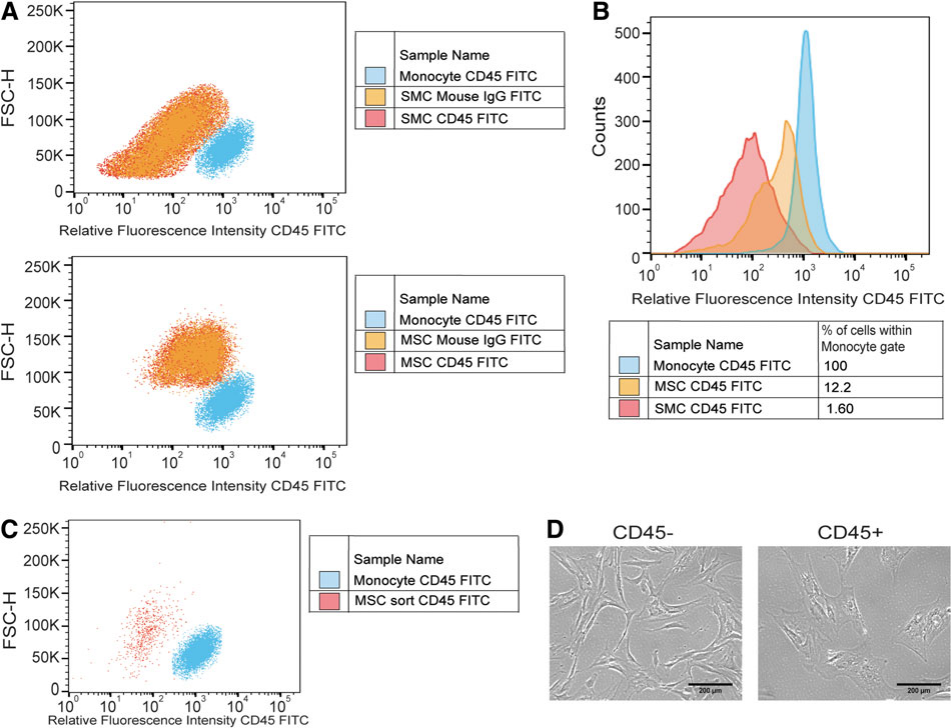
**(A)** Representative dot plot of CD45 expression of SMCs and MSCs relative to monocyte positive controls. FSC-H represents forward scatter as an indicator of cell size. **(B)** Representative histogram demonstrating potential CD45+ cells present within MSC and SMC populations. **(C)** Representation of fluorescently activated cell sorting (FACS) purity analysis after CD45+ cell subtraction in MSC samples **(D)** CD45- and *C*D45+ MSCs after 24 hours of plating. Scale bar equals 200 μm.

We observed the presence of CD45+ contaminating cells within both SMC and MSC populations (Fig. 4A). MSCs contained nearly 8-fold higher amounts of CD45+ cells (12.2%) than SMCs (1.60%) (Fig. 4B). The use of a positive monocyte control for CD45 resulted in an additional 12% of the MSC population gated for negative selection (Fig. 4C). MSCs resulting from sorting displayed unique phenotypes between CD45− and CD45+ eluted populations with the CD45+ conditions resembling a multi-nucleated macrophage phenotype (Fig. 4D).

**FIG. 5.**
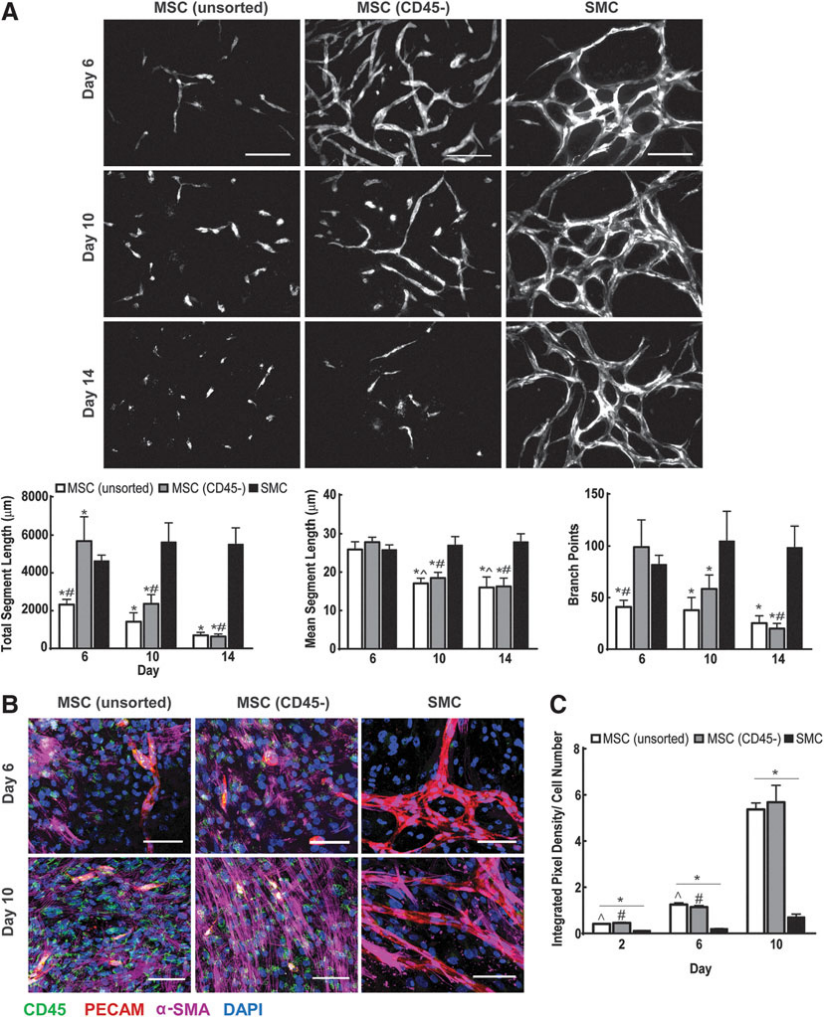
Evaluation of the effect of CD45-expressing cells within MSC populations, upon EOC network formation *in vitro*. **(A)** EOCs were transduced with tomato-expressing lentivirus and combined with either CD45- MSCs, unsorted MSCs, or SMCs for observation over 14 days of coculture. Scale bar equals 200 μm. Changes in EOC network morphology between MSC and SMC coculture conditions over time were quantified through analysis for total segment length, mean segment length, and number of branch points. *p<0.05 in comparison to SMC coculture, ^#^*p*<0.05 in comparison to day 6 MSC (FACS) coculture, ^*p*<0.05 in comparison to day 6 MSC (No FACS). Image area is 0.57 mm^2^, with *n*=8 images analyzed per condition. **(B)** Representative images of immunofluorescence staining for CD45, PECAM, and α-SMA expression at day 6 and day 10 of coculture. Nuclei are shown through DAPI staining. **(C)** CD45 expression was quantified through integrated pixel density and normalized to the total cell number of each image analyzed. *p<0.05 in comparison to SMC coculture, ^#^*p*<0.05 in comparison to day 14 of MSC (FACS) coculture, ^*p*<0.05 in comparison to day 14 of MSC (No FACS). Image area analyzed is 0.65 mm^2^, with *n*=4 images analyzed per condition.

Upon coculture with the CD45-sorted MSC population, EOCs demonstrated significant enhancement in network formation by day 6 in comparison to unsorted MSC cocultures as shown by a 2 fold increase in the total segment length and number of branch points (Fig. 5A). However, the enhancement in EOC networks resulting from CD45- sorting of MSCs was not maintained by 2 weeks of culture, as evidenced through the absence of significant differences in network morphology to unsorted MSCs (*p*=0.5379) (Fig. 5B). To ascertain whether the enhanced EOC network formation in CD45- MSC cocultures was caused by CD45 subtraction rather than an increase in the number of MSCs, we increased the number of unsorted MSCs to 12×10^4^ cells/cm^2^ prior to coculture with EOCs and imaged the resulting network formation over 14 days (Supplementary Fig. S5A, B). However, this 50% increase of MSCs caused no significant enhancement of EOC network throughout the culture period, indicating subtraction of CD45+ cells caused the increase in EOC total segment length and connectivity observed at day 6 of coculture.

Immunofluorescence staining of coculture conditions revealed an increase in CD45 expression in both unsorted and sorted MSC cocultures between day 2 and day 10 of culture in contrast to SMC cocultures (Fig. 5C, D). These findings suggest interaction between EOCs and MSCs may cause proliferation of CD45+ cells, not observed in EOC and SMC interactions, that is associated with EOC network regression and cell loss. CD45+ macrophages have been shown to undergo proliferation during inflammation.^50,51^ Therefore, despite the use of FACS, trace amounts of CD45+ cells may still be present within the CD45− MSC condition, which, upon coculture with EOCs isolated from a separate donor, could be stimulated towards an inflammatory phenotype inflammatory response observed *in vivo*, inducing MSC-macrophage proliferation and phagocytosis of EOCs. While the EOCs contained trace amounts of CD45+ cells (3%–8%) (Supplementary Fig. S6), the presence of CD45+ cells within EPC and SMC cocultures did not lead to the loss of EOC that was observed in the MSC cocultures (Fig. 4A–D).

### The addition of supplemental growth factors prevents EOC network regression and cell loss within MSC cocultures without affecting CD45 expression

**FIG. 6.**
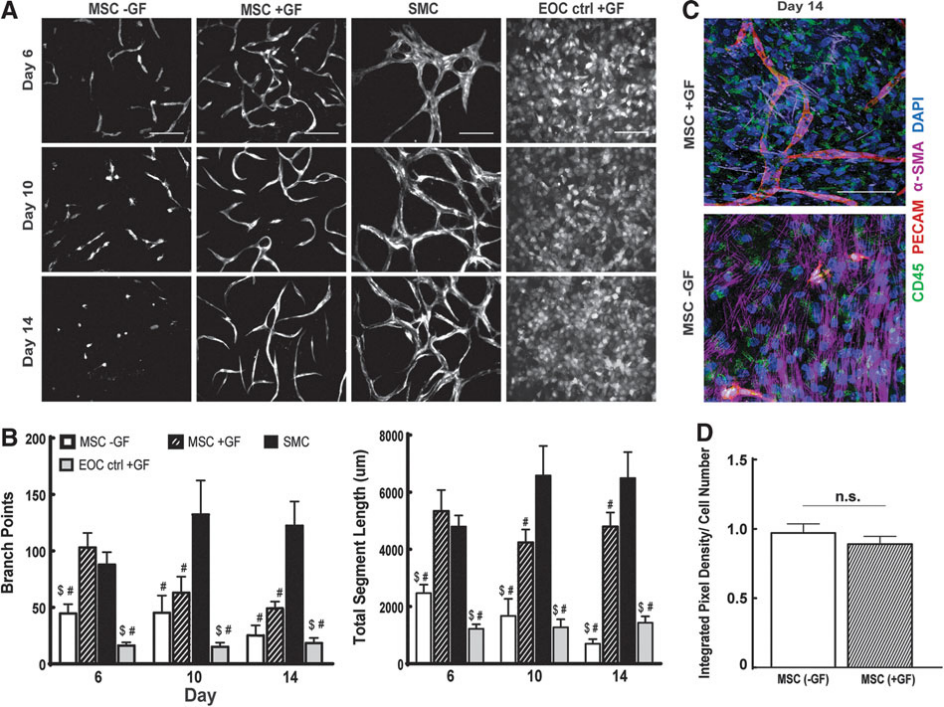
The effect of growth factor addition to unsorted MSC cocultures upon EOC network formation *in vitro*. **(A)** EOCs were transduced with tomato-expressing lentivirus and combined with MSCs culture conditions with supplemental growth factors (+GF) or without (–GF). SMCs cocultures (GF) and EOC monocultures (+GF) were used as controls. **(B)** EOC network morphology was observed over 14 days of coculture and quantified. Scale bar equals 200 μm. ^#^Indicates *p*<0.05 in comparison to SMC coculture, ^$^indicates *p*<0.05 in comparison to MSC (+GF) coculture; image area analyzed is 0.57 mm^2^. **(C)** Representative immunofluorescence staining of MSC (+GF) for CD45, PECAM, and α-SMA expression at day 14 of coculture. Nuclei are shown through DAPI staining. Scale bar equals 100 μm. **(D)** CD45 expression was quantified through integrated pixel density and normalized to the total cell number of each image analyzed. Image area analyzed is 0.65 mm^2^, with *n*=6 images analyzed per condition.

Robust EC network formation previously reported with MSC cocultures employed media conditions containing supplemental angiogenic growth factors.^52,53^ To test the hypothesis that the addition of angiogenic growth factors improves the ability of MSCs to support EOC network formation, we included the full endothelial growth media supplement (EGM2, Lonza) containing VEGF, FGF-2, endothelial growth factor, hydrocortisone, insulin growth factor-1, ascorbic acid, and heparin to cocultures of MSCs and EOCs and observed changes in EOC network morphology over 2 weeks. Addition of these growth factors resulted in robust EOC network formation to levels observed in our positive control, SMC coculture conditions at day 6 of culture and no significant differences in total segment length over time (*p*=0.9603) (Fig. 6A, B). These EOC networks, however, were not as connected as those found in SMC coculture conditions based on the decrease in the number of branch points by day 14 of coculture. Immunofluorescence of EGM2-containing MSC cocultures at day 14 indicates the presence of CD45+ cells (Fig. 6C) were not significantly different from MSC cocultures without EGM2 supplements (p=0.3988) (Fig. 6D), indicating the improved response of EOC networks was not caused by a decrease in CD45+ expression.

## Discussion

Regulation of microvessel development is critical for the healthy function of all organs in the body and the management of several types of disease.^1,2^ One approach for investigating of the role of mural cells upon EC network formation is through tissue engineering, which recapitulates angiogenic processes *in vitro* by means of coculturing vascular cells upon biomaterials under pro-angiogenic conditions.^54^ In addition to serving as a non-invasive research tool for testing novel hypotheses, *in vitro* tissue engineered- microvascular models show promise for direct translation into pro-angiogenic therapies.^7,13,55,56^ Bone marrow–derived MSCs and blood-derived EOCs are promising allogenic sources for tissue-engineered microvessel structures due to their noninvasive isolation and proliferative potential.^8,26,27^

In this study, we evaluated the ability of MSCs to function as SMCs in the support of EOC network formation through the employment of a reductionist, *in vitro* coculture system. Under these conditions, we found coculture of MSCs with EOCs led to EOC loss by day 10 of culture. The cause for EOC loss was not due to a lack of angiogenic cytokines VEGF and TGF-β1, or significant increases in supernatant levels of anti-angiogenic cytokines Ang-2, TSP-1, IFN-γ, and IFN-α. Instead, we found MSC cocultures displayed evidence of phagocytosis, which led us to investigate whether trace amounts of CD45+ cells within MSC populations were the cause of EOC loss. However, subtraction of CD45+ cells within MSCs only led to short-term improvements upon EOC network formation as evidenced through total segment length and number of branch points. The addition of growth factors VEGF, FGF-2, EGF, hydrocortisone, insulin growth factor-1, ascorbic acid, and heparin to MSC cocultures promoted stable EOC network formation over 2 weeks *in vitro*, despite the presence of CD45+ cells, as evidenced by a lack of significant differences in total segment length (*p*=0.96). Further studies are needed to understand whether one, or a combination, of these growth factors is responsible for the enhancement of EOCs network formation upon coculture with MSCs.

The enhancement of EOC networks within MSC cocultures observed upon addition of EGM2 supplements, may be caused by the induction of the potential CD45+ macrophage-like cells towards a neutral or proangiogenic phenotype. This result offers the intriguing hypothesis that a combination of growth factors can be incorporated within a biomaterial scaffold to enable robust, stable microvessel formation of allogenic progenitor cell sources by preventing phagocytosis through the modulation of CD45+ pro-inflammatory cells towards a neutral, or pro-angiogenic state.

## Conclusion

In this study, we observed the presence of CD45+ cells present within MSC populations is correlated with EOC network regression and cell loss. The addition of supplemental growth factors can eliminate this effect. To improve the success of cell-based angiogenic therapies, further studies are needed to determine the macrophage identity of CD45+ cells within MSC populations and investigate their response to angiogenic growth factors with the aim of improving progenitor cell-driven microvessel formation.

## Supplementary Material

Supplemental data

## Abbreviations Used

Ang-2: angiopoietin-2
CSPG: chondroitin sulfate proteoglycan
EBM: endothelial basal media
EC: endothelial cell
EGM2: endothelial growth media supplement
ELISA: enzyme-linked immunosorbent assay
EOC: endothelial outgrowth cell
EPC: endothelial progenitor cell
EthD-1: ethidium homodimer-1
FACS: fluorescently activated cell sorting
FBS: fetal bovine serum
FGF: fibroblastic growth factor
FITC: fluorescein isothiocyanate
GFP: green fluorescent protein
HLA-DR: major histocompatibility complex, class II, DR alpha
IFN: interferon
MNC: mononuclear cells
MSC: mesenchymal stem cells
MYH: myosin heavy chain
PBS: phosphate buffered saline
PDGF: platelet-derived growth factor
PE: phycoerythrin
PECAM: platelet endothelial cell adhesion molecule
SMA: smooth muscle actin
SMC: smooth muscle cell
TGF: transforming growth factor
Tie: tyrosine kinase with immunoglobulin-like and EGF-like domains
TSP: thrombospondin
VEGF: vascular endothelial growth factor
