# Supplementary material for: CD45+ Cells Present Within Mesenchymal Stem Cell Populations Affect Network Formation of Blood-Derived Endothelial Outgrowth Cells
Source: Biores Open Access. 2015 Jan 1;4(1):75–88. doi: 10.1089/biores.2014.0029 (PMC4497669; doi:10.1089/biores.2014.0029)
Supplement: Supplemental data [file Supp_Data.pdf]

Supplementary Materials

Mesenchymal Stem Cell Characterization

Culture

Mesenchymal stem cells (MSCs) were expanded in Minimum Essential Medium Alpha Medium (500 mL, Gibco) supplemented with 100 mL fetal bovine serum (FBS) (Atlanta Biologics), 5 mL penicillin streptomycin solution (100× concentration, Corning), and 5 mL L-Glutamine (200 mM, Lonza). Upon 80% confluence, MSCs were rinsed with Dulbecco's phosphate buffered saline (PBS) (without calcium chloride and magnesium chloride, Sigma) and detached with 0.25% trypsin-EDTA (Gibco) and subcultured at a density of 3000 cells/cm^2. MSCs from two donors were used between passages 3 and 5 for all experiments.

Flow cytometry analysis

MSCs were characterized for positive expression of markers CD90, CD105, CD73, and negative expression of CD45, CD34, CD14, CD19, and HLA-DR in comparison with a mouse immunoglobin G (IgG) isotype control using antibodies preconjugated with fluorescein isothiocyanate (FITC) or phycoerythrin (PE) (Biolegend) at a concentration of 2 μL per 1×10^5 cells. MSCs were detached with 0.25% tryspin–EDTA (Gibco) and centrifuged at 200 g before resuspension in MSC culture media containing antibodies and incubated for 30 minutes at room temperature. The MSCs and antibody mixture underwent a rinsing step with PBS added at an equal volume as the antibody solution and the entire mixture was centrifuged at 200 g for 7 minutes. MSCs were resuspended in 4% paraformaldehyde and stored at 4°C prior to analysis. Analysis was performed using a BD FACSCalibur™ cell analyzer with the minimum number of gated events for analysis as 9000.

Fluorescence-activated cell sorting (FACS) for CD105+/CD45- MSC populations was performed by incubating CD105-PE, CD45-FITC, and mouse IgG isotype controls with detached MSCs at a concentration of 5 μL per 1×10^6 cells for 30 minutes at 4°C. As a rinsing step, culture media was added to each sample and centrifuged at 1000 g for 5 minutes before the media was aspirated and samples resuspended in fresh culture media at a concentration of 1×10^6 cells/mL. Samples were filtered (PARTEC, CellTrics® 30 μm filter) and sorted with BD FACSVantage™ SE flow cytometry system. Eluted cells were captured in MSC culture media, centrifuged at 200 g for 5 minutes, and plated at 3000 cells/cm^2.

Differentiation toward osteogenic, adipogenic, and chondrogenic lineages

StemPro® differentiation kits (Invitrogen) were used to confirm osteogenic, adipogenic, and chondrogenic differentiation of MSCs. Following the manufacturer's suggestions, MSCs were cultured in osteogenic induction media for 24 days, adipogenic induction media for 10 days, and chondrogenic induction media for 15 days before fixation with 10% formalin for one hour. As an indicator of osteoblast mineralized matrix, Alizarin Red (Sigma) was diluted to a concentration of 2 g/mL in distilled water and adjusted to a pH of 4.1 before addition to fixed samples for 2 minutes before rinsing with PBS.

To visualize lipid vacuole formation as an indicator for adipogenic differentiation, Oil Red O at a concentration of 0.3 g/mL in isopropanol and diluted at 3:2 volumetric ratio with distilled water and filtered over Whatman filter paper (Sigma). Adipogenic media-cultured MSCs were incubated with 60% isopropanol for 5 minutes before adding the Oil Red O working solution for 5 minutes. After rinsing twice with PBS, the samples were incubated with Mayer Hematoxylin for 1 minute to visualize nuclei.

To visualize proteoglycans as an indicator for chondrogenic differentiation, Alcian Blue solution (Sigma) was prepared at 1% concentration in 0.1 N hydrochloric acid and added to fixed samples for 30 minutes before rinsing with distilled water. The resulting immunohistochemical samples were captured under light microscope conditions with a Nikon® Eclipse Inverted Microscope system

Endothelial Outgrowth Cell Isolation and Characterization

Umbilical cord blood was obtained from the Carolina Cord Blood Bank at Duke University following approval from the Duke University Institutional Review Board. Endothelial progenitor cells were isolated using a protocol previously established by Ingram et al.[26] Whole blood containing 35%–50% anticoagulant was diluted 1:1 with Hank's Balanced Salt Solution

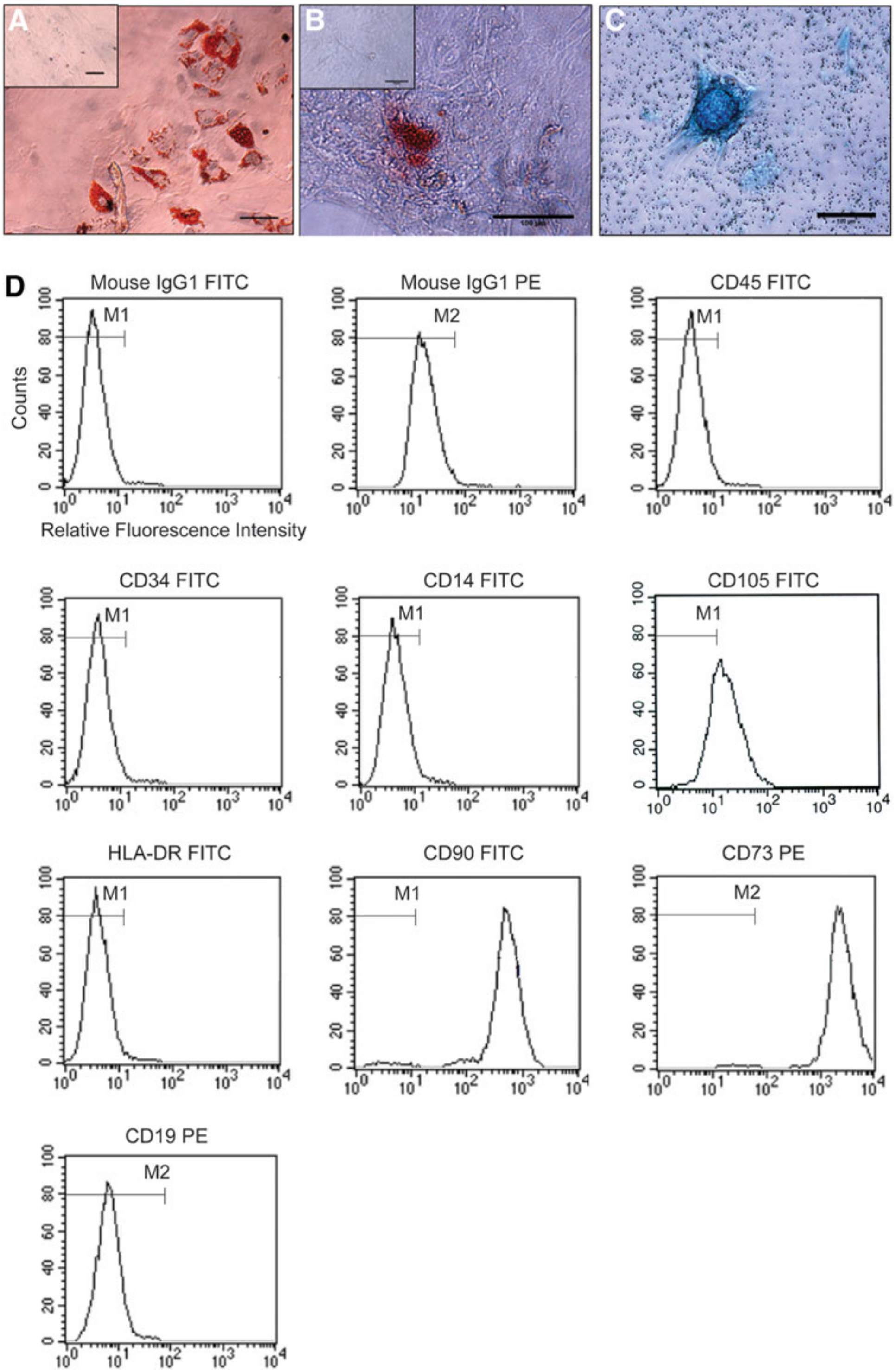

SUPPLEMENTARY FIG. S1. Differentiation of mesenchymal stem cells MSCs into **(A)** adipogenic, **(B)** osteogenic, and **(C)** chondrogenic lineages. Insets **(A, B)** represents control conditions. Scale bar equals 100 μm. **(D)** Representative histograms from MSC flow cytometry analysis.

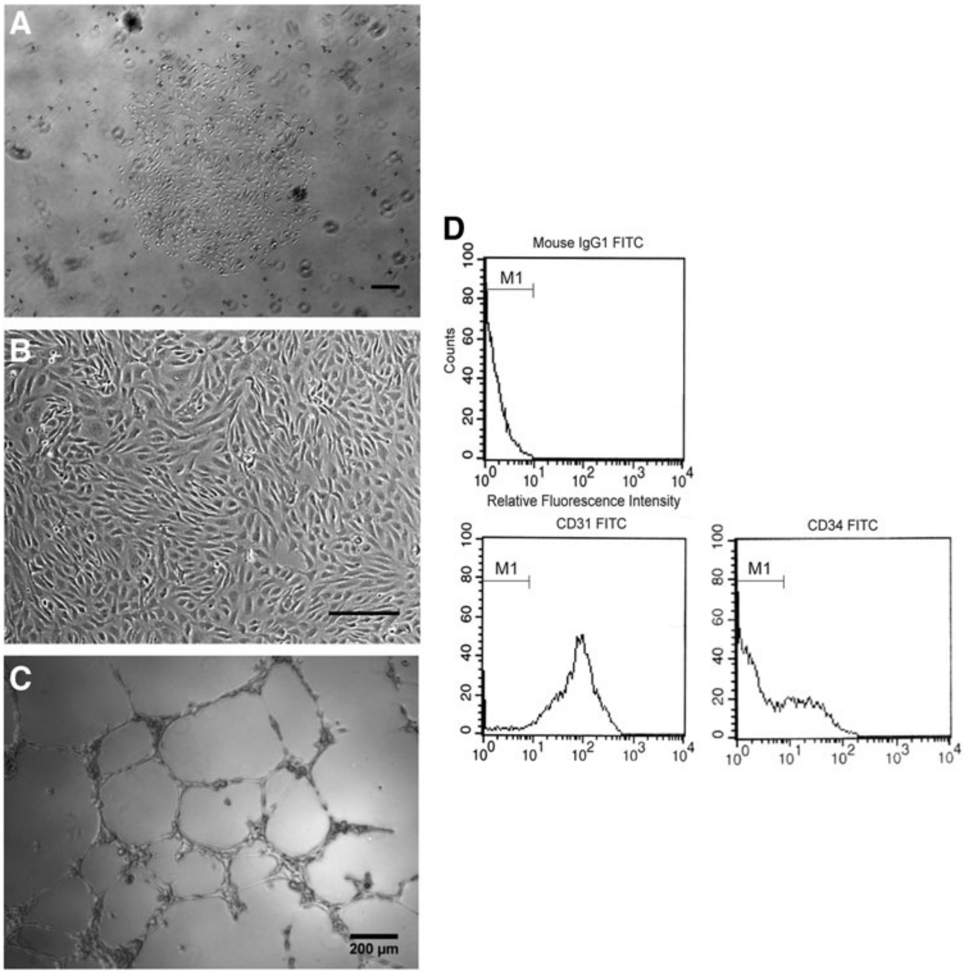

SUPPLEMENTARY FIG. S2. **(A)** Representative images of endothelial colony-forming units after 9 days of plating mononuclear cells. **(B)** Endothelial outgrowth cells (EOCs) expanded from endothelial colony-forming units. Scale bar equals 100 μm. **(C)** EOC network formation within Matrigel™ 6 hours after initial plating. Scale bar equals 200 μm. **(D)** Representative histograms from EOC flow cytometry analysis.

(Gibco) before gentle layering atop Histopaque-1077 (Sigma) solution. The Histopaque and blood mixture were centrifuged at 750 *g* for 30 min to enable isolation of monocular cells (MNCs). The resulting MNC layer was removed and underwent 3 rinse cycles with endothelial basal media-2 (EBM-2, Lonza) supplemented with endothelial growth media-2 SingleQuots (EGM-2, Lonza) containing vascular endothelial growth factor 165 (0.09% v/v) human epidermal growth factor (0.09% v/v), hydrocortisone (0.36% v/v), gentamicin (0.09% v/v), human recombinant fibroblastic growth factor-beta (0.36% v/v), insulin-like growth factor (0.09% v/v), abscorbic acid (0.09% v/v), and heparin (0.09% v/v), a total of 50 mL of FBS (8.9% v/v), and 5 mL of penicillin streptomycin solution (0.89% v/v). Each rinse cycle lasted 10 min at 515 *g*. MNCs were plated at a density of 80×10^6 cells/well onto a 6-well plates (BD Falcon) pre-coated for one hour with 50 μg/mL of rat tail collagen I (BD Biosciences). Endothelial colony-forming units (ECFU) were observed 9–22 days after MNC plating.

Culture

ECFUs were passaged with 0.025% trypsin-EDTA and seeded onto T-25 flasks supplemented with EBM-2 media supplemented with EGM-2, a total of 50 mL of FBS, and 5 mL of penicillin streptomycin solution.

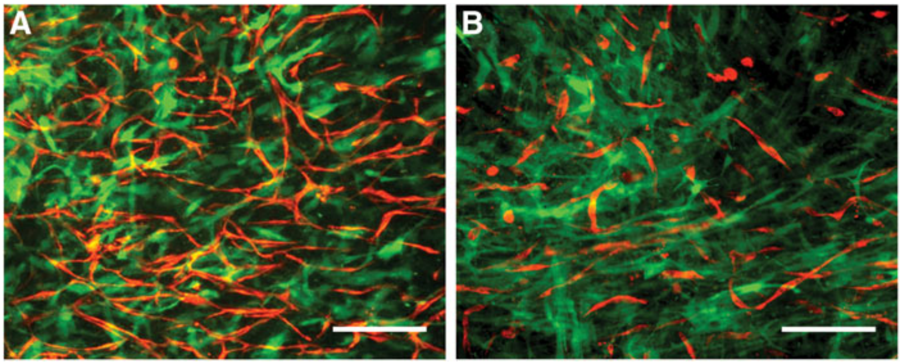

SUPPLEMENTARY FIG. S3. Representative images of EOCs (red) at day 10 of coculture with **(A)** smooth muscle cells (SMCs) (green) and **(B)** MSCs (green). Scale bar equals 250 μm.

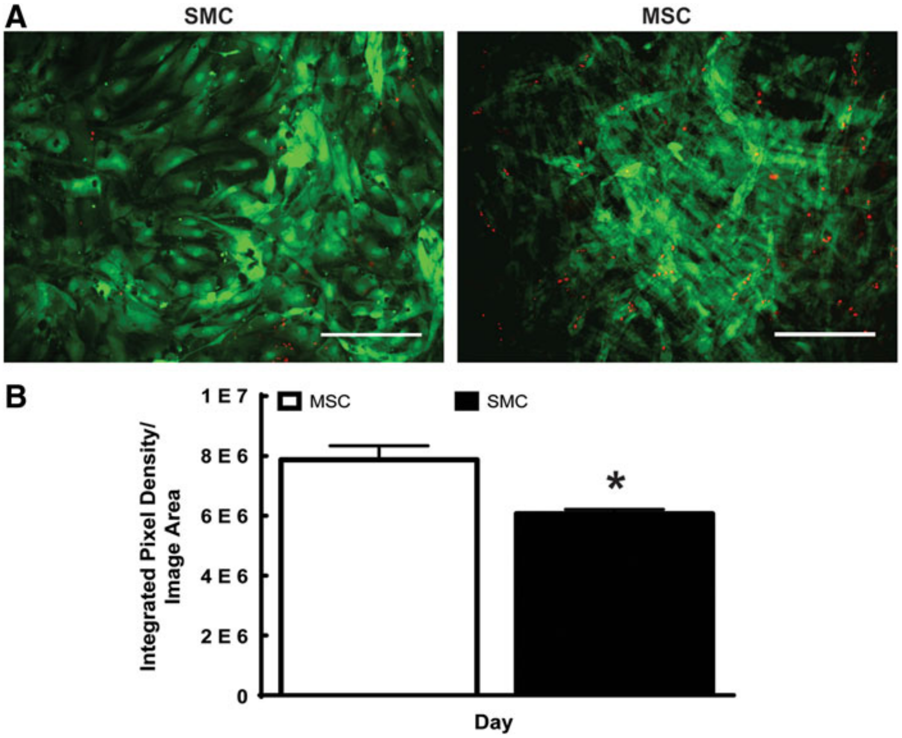

SUPPLEMENTARY FIG. S4. **(A)** Representative images depicting EOC viability within MSC and SMC cocultures through the absence of ethidium homodimer-1 (EthD-1) expression (red) after 8 days of culture. Live cells are depicted through calcein AM expression (green). Scale bar equals 200 μm. **(B)** Quantification of EthD-1 expression after 8 days of EOCs in coculture with MSCs or SMCs. * Indicates $p < 0.05$ in comparison with SMC coculture condition. Four images analyzed per condition. Image area analyzed is 0.57 mm^2.

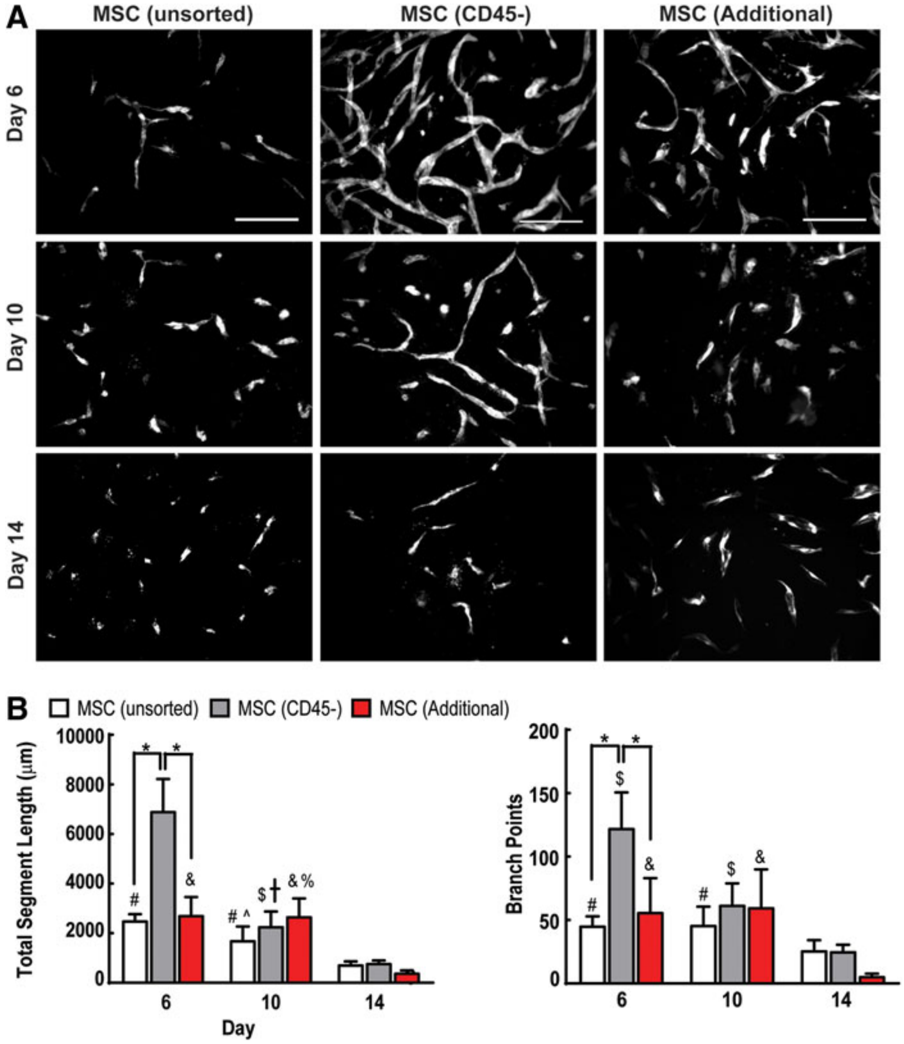

SUPPLEMENTARY FIG. S5. Evaluation of the effect of increasing MSC amounts upon EOC network formation. **(A)** Representative images of EOCs transduced with tomato-expressing lentivirus and combined at 4.8×10^4 cells/cm^2 with 12×10^4 cells/cm^2 unsorted MSCs (MSC additional), 8×10^4 cells/cm^2 of unsorted MSCs (MSC unsorted), or 8×10^4 cells/cm^2 of MSCs subtracted for CD45+ populations (MSC CD45–). Scale bar equals 200 μm. **(B)** Quantification of EOC networks for total tubule length and number of branch points among MSC coculture conditions. *Indicates $p < 0.05$, $^{\#}$indicates $p < 0.05$ in comparison with day 14 of MSC (unsorted) coculture, ^indicates $p < 0.05$ in comparison with day 6 of MSC (unsorted) coculture, $^{\$}$indicates $p < 0.05$ in comparison with day 14 of MSC (CD45-) coculture, †indicates $p < 0.05$ in comparison with day 6 of MSC (CD45-) coculture, $^{\&}$indicates $p < 0.05$ in comparison with day 14 of MSC (additional) coculture, $^{\%}$indicates $p < 0.05$ in comparison with day 6 of MSC (additional) coculture. Six images were analyzed per condition. Image area analyzed is 0.57 mm^2.

The resulting EOCs were passaged upon 80% confluence with 0.25% trypsin-EDTA and re-plated at a density of 6000 cells/cm^2. EOCs were derived from 3 independent donors and used between passages 3 and 5 for all experiments.

Flow cytometry analysis

To confirm endothelial outgrowth cell (EOC) phenotype of expanded ECFUs, flow cytometry was performed for expression of endothelial cell marker platelet-derived cell adhesion molecule (PECAM or

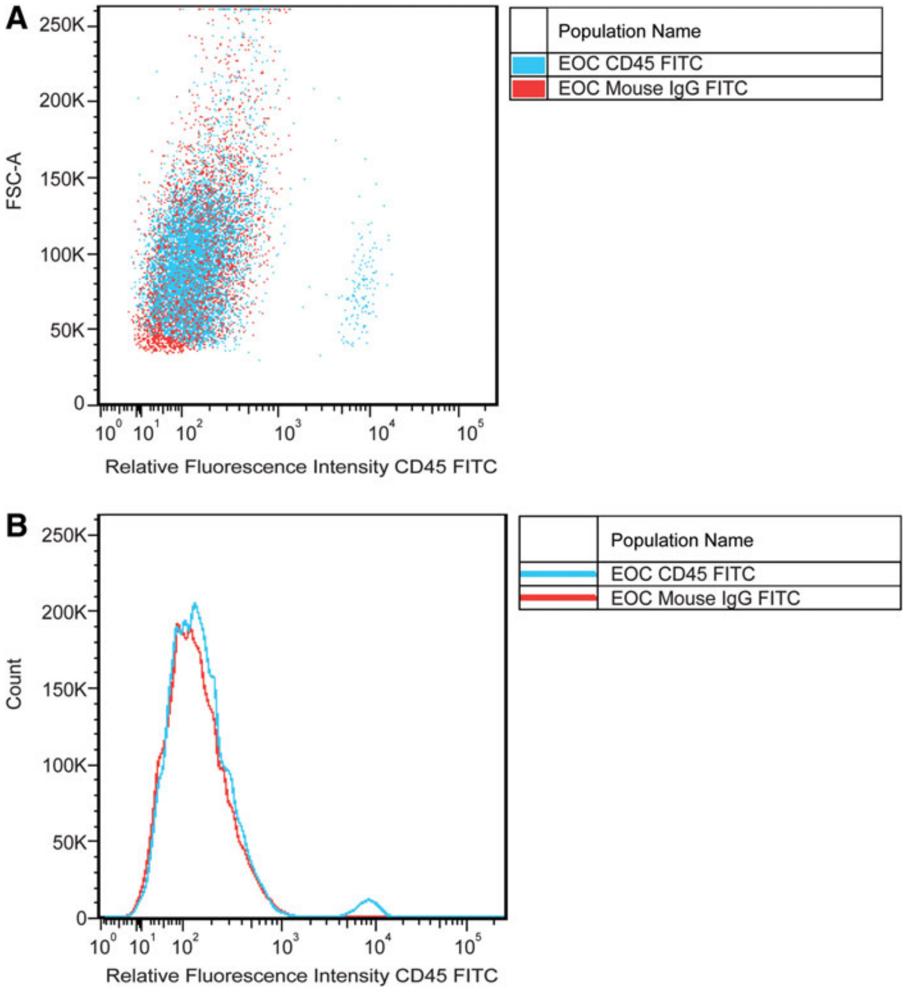

SUPPLEMENTARY FIG. S6. Representative dot plot **(A)** and histogram **(B)** from flow cytometry analysis of EOCs at passage 2 that demonstrates trace populations (3%) of CD45+ cells.

CD31) and hematopoietic stem cell marker CD34, expression of lymphocyte marker CD45 in comparison to a mouse IgG isotype control using antibodies preconjugated with FITC or PE (Biolegend) following conditions used for MSC flow cytometry characterization.

Matrigel™ network formation assay

To confirm the angiogenic potential of EOCs prior to co-culture with MSCs and smooth muscle cells, Matrigel® basement membrane matrix (Corning) was thawed from −20°C aliquots overnight, on ice, at 4°C, before

Supplementary Table S1. Primers for Quantitative PCR Analysis

Gene	Forward primer (5′–3′)	Reverse primer (5′–3′)
VEGFR-2	CCT GCC ATC TGA TGA ATT CTG TCC C	TGG GGA GCA AAA CCT TCC TCT TCT A
CSPG-4	GTC CGA CGG GCA ACA CCA GG	GCC ACG CGA CAC CAT CAC CA
PDGFβ-R	TGC CCC GTC CAA CAA CAC GG	CCT CCA GTG GGC CCT CGT CA
MYH-11	GCA GTG GAT AAG CTC TTC CCA ACC C	CCT GAG CAG ACG CTG CCA CAT
TIE-1	GCT GCA TGG CCG GTG TTG AGA A	GGC TTT GGG CTC CGA TGG CA
TIE-2	CTG CGG CCA CAT GAC CTC ACA AT	CAG CGT GGT GGC ACT GCA TC
18s rRNA	CCG CTT TCT GCC GAG ATG CC	GCT GCC CAA TCC CCG TGT TG

CSPG, chondroitin sulfate proteoglycan-4; *MYH-11*, myosin heavy chain 11; PDGFβ-R, platelet-derived growth factor β receptor; VEGFR-2, vascular endothelial growth factor receptor 2; TIE, tyrosine kinase with immunoglobulin-like and EGF-like domains.

addition to 8-well chamber slides at a volume of 125 μL per well, over ice, and allowed to polymerize to form gels for 30 min in a cell culture incubator at 37°C under 5% CO_2. EOCs were plated at a seeding density of 4×10^4 cells/mL with EBM2 containing 10 mL of FBS and 5 mL of penicillin-streptomycin. Images were taken 6 hours after the addition of cells to the Matrigel™ matrix.

Monocyte Culture

Human monocytes were purchased from ATCC and maintained in suspension culture with RPMI-1640 (ATCC) growth media containing 50 mL of FBS, 5 mL of penicillin streptomycin solution, and 0.05 mM of 2-mercaptoethanol. Media was changed every 48 hours to monocytes by centrifuging cells at 200g for 7 min with resuspension at a concentration of 1×10^6 cells/mL. Monocytes were then processed for flow cytometry analysis using CD45-FITC and mouse IgG isotype-FITC following identical procedures as MSCs.

Quantitative PCR Analysis

Total RNA was extracted from cultures containing both EOCs and MSCs using Aurum™ Total RNA Mini Kit (Bio-Rad). RNA purity and quantity were measured using a NanoDrop Spectrophotometer (NanoDrop Technologies). Total RNA (50 ng) was reverse transcribed using the cDNA Synthesis Kit (Bio-Rad) and a MyCycler (Bio-Rad) thermal cycler. One cycle of 5 min at 25°C, 30 min at 43°C, and 5 min at 85°C was performed. Primers (Integrated DNA Technologies), RNAse free water, and IQ SYBR Green Supermix (Bio-Rad) were combined with the cDNA samples and placed in a MyIQ Single Color Real-Time PCR Detection System (Bio-Rad). A two-step cycle configuration was performed with an initial denaturation for 3 min at 95°C and 50 cycles at 95°C for 15s and 61°C for 1 min. All samples were performed in triplicate for all genes. The 2(-Delta Delta C(T)) method was used to determine relative gene expression to smooth muscle cells. Primers were selected based on the gene sequence using the National Center for Biotechnology Information catalogue. Those sequences reviewed and validated in literature were entered into PrimerBLAST designing tool software. To assess primer specificity to targets, expected product bands were confirmed using conventional PCR. The sequences for each primer are listed in Supplementary Table S1.

Immunofluorescence

To further characterize EOC and MSC cocultures, immunofluorescence for protein expression of alpha smooth-muscle actin, PDGFβ-R, CD45, and PECAM/ CD31 was performed on MSC and endothelial progenitor cell cocultures. Cocultures were plated on 35 mm glass dishes (FluoroDishTM, World Precision Instruments, Inc.), which had been incubated with 3.3 μg/mL of fibronectin for one hour. Cocultures were fixed in 4% paraformaldehyde for 10 minutes before rinsing 3 times with PBS. Cultures were blocked in a 3.5% solution of bovine serum albumin (BSA) in PBS for a minimum of one hour. Primary antibodies were diluted with blocking solution at the following concentrations: alpha smooth-muscle actin 1:100 (rabbit polyclonal and mouse monoclonal anti-human, Abcam), platelet-derived growth factor receptor beta (PDGFR-β) 1:50 (rabbit polyclonal anti-human, Santa Cruz), CD45 1:500 (rabbit polyclonal anti-human, Abcam), PECAM 1:100 (mouse monoclonal anti-human, Invitrogen, and goat polyclonal anti-human, Santa Cruz) and incubated with the cultures at 4°C overnight. Samples were washed twice with PBS containing 0.01% Tween-20 (Sigma) for 2 h each rinse and PBS for 2 h before incubation with the appropriate secondary antibody [1:200 dilution in 3.5% bovine serum albumin in PBS of donkey anti-mouse/ donkey anti-rabbit/donkey anti-goat Alexa Fluor 488, 555, or 647 (Invitrogen)] overnight at 4°C. In order to visualize nuclei, cultures were rinsed with PBS and incubated with 5 μg/mL DAPI solution for 1 h. The cultures were rinsed twice with PBS and imaged on a Leica SP5 confocal microscope at 20X magnification. Images were taken at 1280 × 1280 resolution with a line scanning average of 6.
